# Rheumatoid arthritis T cell and muscle oxidative metabolism associate with exercise-induced changes in cardiorespiratory fitness

**DOI:** 10.1038/s41598-022-11458-4

**Published:** 2022-05-06

**Authors:** Brian J. Andonian, Alec Koss, Timothy R. Koves, Elizabeth R. Hauser, Monica J. Hubal, David M. Pober, Janet M. Lord, Nancie J. MacIver, E. William St Clair, Deborah M. Muoio, William E. Kraus, David B. Bartlett, Kim M. Huffman

**Affiliations:** 1 Division of Rheumatology and Immunology, Duke University School of Medicine, Durham, NC 27701 USA; 2 Duke Molecular Physiology Institute, Duke University School of Medicine, Durham, NC 22701 USA; 3 Department of Kinesiology, Indiana University-Purdue University Indianapolis School of Health & Human Sciences, Indianapolis, IN 46202 USA; 4 Phastar Inc, Cambridge, MA USA; 5 MRC-Versus Arthritis Centre for Musculoskeletal Ageing Research, Institute of Inflammation and Ageing, University of Birmingham, Birmingham, UK; 6 NIHR Birmingham Biomedical Research Centre, University Hospital Birmingham and University of Birmingham, Birmingham, UK; 7 Department of Pediatrics, University of North Carolina, Chapel Hill, NC 27514 USA; 8 Faculty of Health and Medical Sciences, University of Surrey, Guildford, UK

**Keywords:** Rheumatoid arthritis, Energy metabolism

## Abstract

Rheumatoid arthritis (RA) T cells drive autoimmune features via metabolic reprogramming that reduces oxidative metabolism. Exercise training improves cardiorespiratory fitness (i.e., systemic oxidative metabolism) and thus may impact RA T cell oxidative metabolic function. In this pilot study of RA participants, we took advantage of heterogeneous responses to a high-intensity interval training (HIIT) exercise program to identify relationships between improvements in cardiorespiratory fitness with changes in peripheral T cell and skeletal muscle oxidative metabolism. In 12 previously sedentary persons with seropositive RA, maximal cardiopulmonary exercise tests, fasting blood, and *vastus lateralis* biopsies were obtained before and after 10 weeks of HIIT. Following HIIT, improvements in RA cardiorespiratory fitness were associated with changes in RA CD4 + T cell basal and maximal respiration and skeletal muscle carnitine acetyltransferase (CrAT) enzyme activity. Further, changes in CD4 + T cell respiration were associated with changes in naïve CD4 + CCR7 + CD45RA + T cells, muscle CrAT, and muscle medium-chain acylcarnitines and fat oxidation gene expression profiles. In summary, modulation of cardiorespiratory fitness and molecular markers of skeletal muscle oxidative metabolism during exercise training paralleled changes in T cell metabolism. Exercise training that improves RA cardiorespiratory fitness may therefore be valuable in managing pathologically related immune and muscle dysfunction.

Trial registration: ClinicalTrials.gov, NCT02528344. Registered on 19 August 2015.

## Introduction

Rheumatoid arthritis (RA) is a systemic autoimmune disease that is associated with low exercise tolerance, disability, and increased risk for cardiovascular disease and mortality^1–7^. Even when in disease remission, RA patients continue to have altered body composition (i.e., high fat mass, low muscle mass), low muscle strength and decreased physical function, implicating persistent systemic metabolic deficits^6,8^.

Oxidative metabolism impairments are observed across multiple tissues in RA that include skeletal muscle and immune cells. For example, the RA skeletal muscle metabolic phenotype is marked by: (1) greater pro-inflammatory cytokine concentrations and canonical NF-κB signaling; (2) greater glycolytic flux with an accumulation of pyruvate; and (3) poorer efficiency for oxidative phosphorylation and ATP generation^9^. Similarly, RA naïve CD4 + T cells undergo metabolic reprogramming consistent with a shift from glycolysis to the anabolic pentose phosphate pathway and reduced oxidative metabolism and ATP production in favor of adipogenesis; these changes lead to T cells marked by hyperproliferation, hypermigration, and tissue invasiveness^10–12^. Though immunometabolic defects and physical inactivity are implicated in RA pathogenesis^12,13^, the relationships among muscle, immune, and systemic metabolic factors is unclear. Cardiorespiratory fitness—as assessed by maximal oxygen consumption at peak exercise (i.e., VO_2_ peak)—is a measure of systemic oxidative metabolism and the ability to transport and utilize oxygen in mitochondria to perform the work of physical activity^14^. Since decreased cardiorespiratory fitness is one of the strongest predictors of cardiovascular disease and all-cause mortality, management of RA requires breaking the vicious cycle that links physical inactivity with impaired oxidative metabolism^14,15^.

We previously observed that high-intensity interval training (HIIT)—alternating one-minute bouts of high- and low-intensity aerobic exercise for 30 min per session on three days per week for 10 weeks—significantly increased cardiorespiratory fitness (as measured by relative VO_2_ peak) in patients with RA by 9% on average but with significant heterogeneity among patients in their response^16^. HIIT also improved skeletal muscle remodeling and innate immune cell inflammatory function in these patients^16,17^. We also observed that HIIT improved RA inflammatory disease activity in association with baseline transcriptomic remodeling of skeletal muscle amino acid and oxidative metabolism, providing evidence for a key role of skeletal muscle metabolism in reversing RA immune dysfunction^18^. While exercise training and increasing physical activity increase immune cell mitochondrial oxidative metabolism in healthy young and elderly individuals^19–23^, to our knowledge, this effect of exercise has not been previously studied in patients with RA.

Here, we sought to expand our understanding of the effects of exercise training on RA beyond previous findings that HIIT improves RA cardiorespiratory fitness and disease activity. Using stored samples from the previously completed study^16–18^, we completed new analyses of changes in RA T cell metabolism and biomarkers of skeletal muscle metabolism following HIIT. Thus, the goal of this study was to take advantage of the heterogenous effects of exercise training on cardiorespiratory fitness to identify relationships between changes in RA systemic and organ-specific metabolism in the HIIT-trained cohort.

## Results

### HIIT improves cardiorespiratory fitness in association with changes in RA CD4 + T cell oxidative function

**Figure 1:**
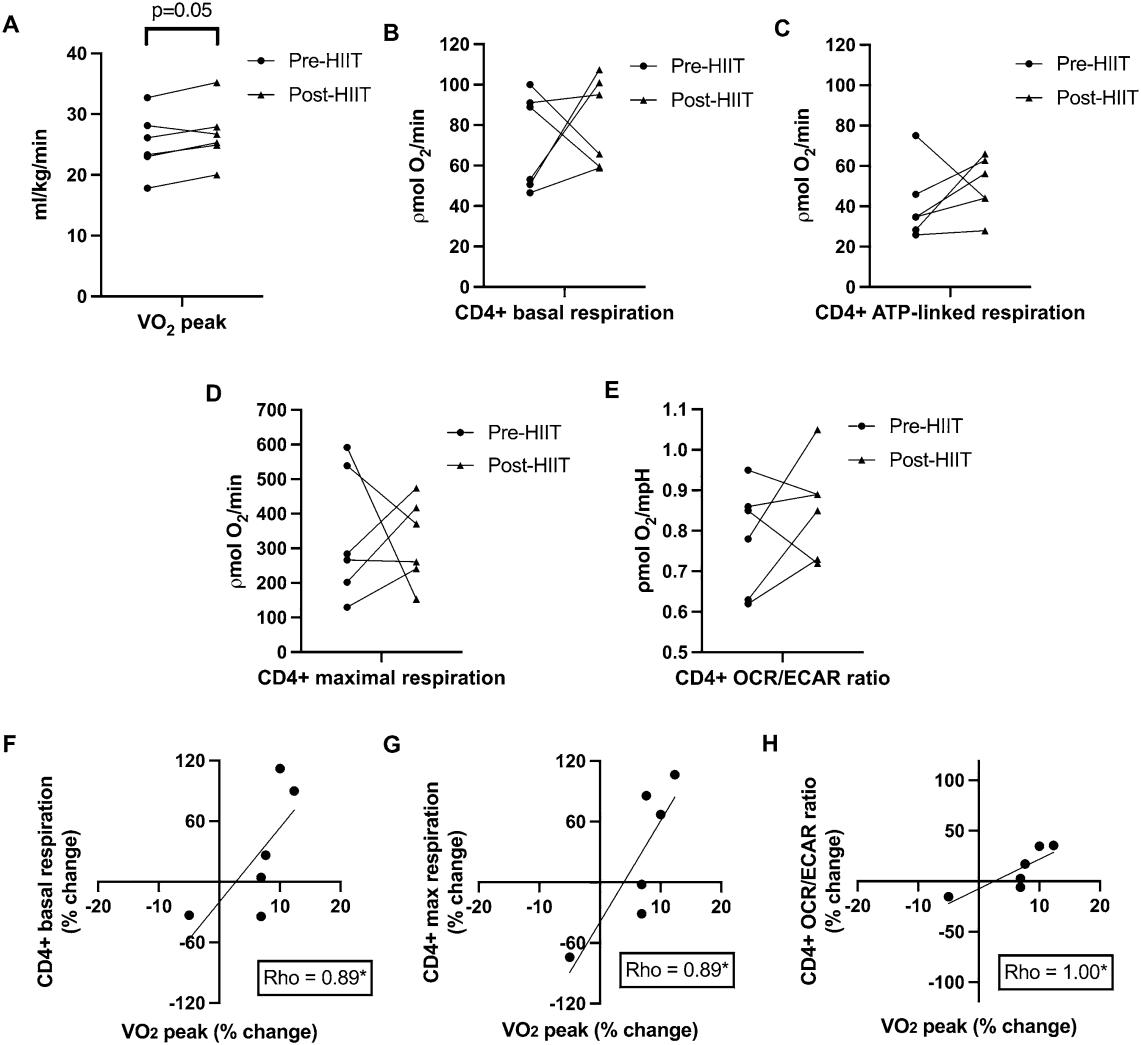
HIIT improves cardiorespiratory fitness in association with changes in RA CD4 + T cell oxidative function. Graphs show changes from before (Pre-HIIT) to after (Post-HIIT) high-intensity interval training (HIIT) in individual rheumatoid arthritis (RA) participant (subgroup n = 6) (**A**) cardiorespiratory fitness (relative VO_2_; ml/kg/min) and peripheral CD4 + T cell (**B**) basal, (**C**) ATP-linked, and (**D**) maximal respiration (oxygen consumption rate; ρmol O_2_/minute), and (**E**) basal oxygen consumption rate (OCR)/extracellular acidification rate (ECAR) ratio (OCR/ECAR ratio; ρmol/mpH). (**F**) Scatter plot depicts relationship between percent change in RA peripheral CD4 + T cell basal respiration (y-axis) and percent change in relative peak VO_2_ (x-axis) following HIIT. (**G**) Scatter plot depicts relationship between percent change in RA peripheral CD4 + T cell maximal respiration (y-axis) and percent change in relative peak VO_2_ (x-axis) following HIIT. (**H**) Scatter plot depicts relationship between percent change in RA CD4 + T cell OCR/ECAR ratio (y-axis) and percent change in relative peak VO_2_ (x-axis) following HIIT. *p < 0.05 for paired t-tests and Spearman correlations.

We found that persons with RA significantly increased systemic oxidative capacity, or cardiorespiratory fitness (measured as VO_2_ peak via cardiopulmonary exercise testing), following 10-weeks of HIIT (n = 12; mean pre-HIIT relative VO_2_ peak = 25.0 ml/kg/min, SD = 6.1; mean post-HIIT relative VO_2_ peak = 27.1 ml/kg/min, SD = 7.0, p < 0.001, Cohen’s *d* = 3.19) (Table 1)^16^. Of the 12 RA participants who completed the HIIT program where peripheral immune cell and skeletal muscle biopsy samples were used for initial primary analyses^16,17^, 6 RA participant immune cell (i.e., T cell) samples and 9 RA participant skeletal muscle samples remained for further pilot-study asssessments. RA participants from T cell (Fig. 1A) and skeletal muscle (Fig. 3A) subgroups from the larger cohort also increased cardiorespiratory fitness following HIIT. Given this finding, we hypothesized that improved fitness from exercise training would lead to increased oxidative metabolic function in individual organ systems and cell types. Our primary interest was to assess the effects of HIIT on CD4 + T cells given the importance of altered CD4 + T cell metabolic function in contributing to the pathogenesis and perpetuation of RA^24^. As measured by the Seahorse XF Mito Stress Test from available peripheral blood lymphocytes samples from the small subgroup (n = 6 from the larger cohort of 12 RA participants), there were no significant mean changes in isolated RA CD4 + T cell oxidative (oxygen consumption rate (OCR); ρmol/min) (Fig. 1B-D) or basal glycolytic (extracellular acidification rate (ECAR); mpH/min) metabolism following HIIT (Supplemental Fig. 1). However, RA CD4 + T cells obtained before and after training preferred glycolytic metabolism (pre-HIIT mean basal OCR/ECAR ratio = 0.78 ρmol/mpH, SD = 0.13; post-HIIT mean basal OCR/ECAR ratio = 0.86 ρmol/mpH, SD = 0.16, p = 0.30) (Fig. 1E).

**Table 1:** Rheumatoid arthritis patient clinical characteristics.

Variable	HIIT RA cohort (n = 12)	HIIT RA cohort: T cell subgroup (n = 6 of 12)	HIIT RA cohort: Skeletal muscle subgroup (n = 9 of 12)
Age, mean years (SD)	63.9 (7.2)	64.0 (6.3)	63.0 (7.5)
**Gender, n (%)**			
Female	11 (91.6%)	6 (100%)	8 (88.9%)
Male	1 (8.4%)	0 (0%)	1 (11.1%)
Rheumatoid factor positive, n (%)	10/12 (83.3%)	5/6 (83.3%)	7/9 (77.8%)
Anti-cyclic citrullinated antibody positive, n (%)	5/8 (62.5%)	0/2 (0%)	4/6 (66.7%)
Erosions on radiographs present, n (%)	9/12 (75.0%)	5/6 (83.3%)	6/9 (66.7%)
Disease duration, months (SD)	159.6 (86.7)	160.0 (108.3)	140.0 (88.0)
**DAS-28, mean (SD)**			
Baseline	3.1 (1.5)	2.6 (0.4)	2.8 (1.0)
Post-HIIT	2.3 (1.5)*	1.9 (0.5)*	2.1 (0.8)*
**BMI, mean kg/m**^**2**^** (SD)**			
Baseline	27.4 (9.3)	26.0 (4.2)	24.7 (4.8)
Post-HIIT	27.7 (9.8)	26.0 (4.2)	24.8 (4.8)
**Cardiorespiratory fitness (VO**_**2**_** peak), mean ml/kg/min (SD)**			
Baseline	24.9 (6.6)	25.2 (5.1)	27.6 (4.2)
Post-HIIT	27.1 (7.0)*	26.7 (5.0)^#^	29.8 (4.6)*
**RA medication use, n (%)**			
Infliximab	2 (16.7%)	1 (16.7%)	2 (22.2%)
Adalimumab	2 (16.7%)	1 (16.7%)	2 (22.2%)
Tofacitinib	1 (8.3%)	1 (16.7%)	1 (11.1%)
Methotrexate	6 (50%)	3 (50%)	3 (33.3%)
Leflunomide	1 (8.3%)	1 (16.7%)	1 (11.1%)
Sulfasalazine	2 (16.7%)	0 (0%)	2 (22.2%)
Hydroxychloroquine	4 (33.3%)	1 (16.7%)	3 (33.3%)
NSAIDs	8 (66.7%)	2 (33.3%)	7 (77.8%)
Prednisone (< 5 mg/day)	3 (25%)	1 (16.7%)	3 (33.3%)

HIIT high intensity interval training, RA rheumatoid arthritis, DAS-28 disease activity score in 28 joints, BMI body mass index.

*p < 0.05 for paired t-test comparisons between pre-HIIT and post-HIIT.

^#^p = 0.05 for paired t-test comparisons between pre-HIIT and post-HIIT.

We then asked if these variable HIIT responses in peripheral T cell respiration were correlated with changes in cardiorespiratory fitness. Following HIIT, changes in RA cardiorespiratory fitness were significantly associated with changes in RA peripheral CD4 + T cell basal and maximal respiration (Spearman’s rho = 0.89, p = 0.02 for both; Fig. 1F-G) and OCR/ECAR ratio (rho = 1.00, p < 0.001; Fig. 1H).

In summary, after HIIT, RA CD4 + T cells on average did not increase utilization of oxidative pathways, however, changes in T cell metabolism were strongly associated with HIIT-induced changes in cardiorespiratory fitness.

### Changes in RA T cell oxidative metabolism following HIIT are associated with changes in naïve T cells

**Figure 2:**
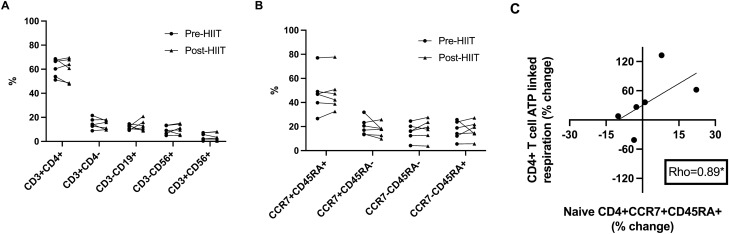
Changes in RA T cell oxidative metabolism following HIIT are associated with changes in naïve T cells. (**A**) Graphs show changes in rheumatoid arthritis (RA) peripheral CD3 + CD4 + helper T cells, CD3 + CD4- non-helper T cells, CD3-CD19 + B cells, CD3-CD56 + natural killer cells, and CD3 + CD56 + natural killer T cells following high-intensity interval training (HIIT) (subgroup n = 6; p > 0.05 for all pre-HIIT versus post-HIIT comparisons). (**B**) Graphs show changes in RA peripheral naïve CCR7 + CD45RA + , central memory CCR7 + CD45RA-, effector memory CCR7-CD45RA-, and terminally differentiated CCR7-CD45RA + CD4 + T cells following HIIT (p > 0.05 for all pre-HIIT versus post-HIIT comparisons). (**C**) Scatter plot depicts relationship between percent change in RA peripheral CD4 + T cell ATP linked respiration (y-axis) and percent change in peripheral naïve CD4 + T cells (x-axis) following HIIT. *p < 0.05 for paired t-tests and Spearman correlations.

In non-RA populations, cardiorespiratory fitness and exercise training are linked to decreased proportions of inflammatory and senescent peripheral lymphocyte and T cell populations^25–27^. Given that low numbers of circulating naïve T cells and high numbers of circulating terminally differentiated T cells are markers of immunosenesence^28,29^, we hypothesized that in RA, HIIT-related T cell oxidative function changes would be related to changes in naïve and terminally differentiated T cells. Absolute lymphocyte numbers (mean pre-HIIT = 2,270 cells/mL, SD = 0.65; mean post-HIIT = 2,230 cells/mL, SD = 0.55, p = 0.83) were unchanged with HIIT, while flow cytometry analyses showed lymphocyte subset frequencies were not different following HIIT (Fig. 2A). Similarly, the frequency of CD4 + T cell subpopulations—including naïve (CD4 + CCR7 + CD45RA +) and terminally differentiated (CD4 + CCR7-CD45RA +) T cells—did not significantly change after HIIT (p > 0.05 for all; Fig. 2B). However, similar to CD4 + T cell metabolic responses, there was large inter-individual subpopulation variability in circulating T lymphocyte numbers and frequencies. Consistent with our hypothesis, HIIT-related changes in T cell ATP-linked respiration were positively correlated with changes in the numbers of naïve T cells (n = 6; rho = 0.89, p = 0.02) (Fig. 2C); however, changes in T cell ATP-linked respiration were not correlated with changes in terminally differentiated T cells (n = 6; rho = − 0.08, p = 0.87).

### HIIT increases RA skeletal muscle carnitine acetyltransferase enzyme activity in association with increased cardiorespiratory fitness and changes in CD4 + T cell oxidative function

**Figure 3:**
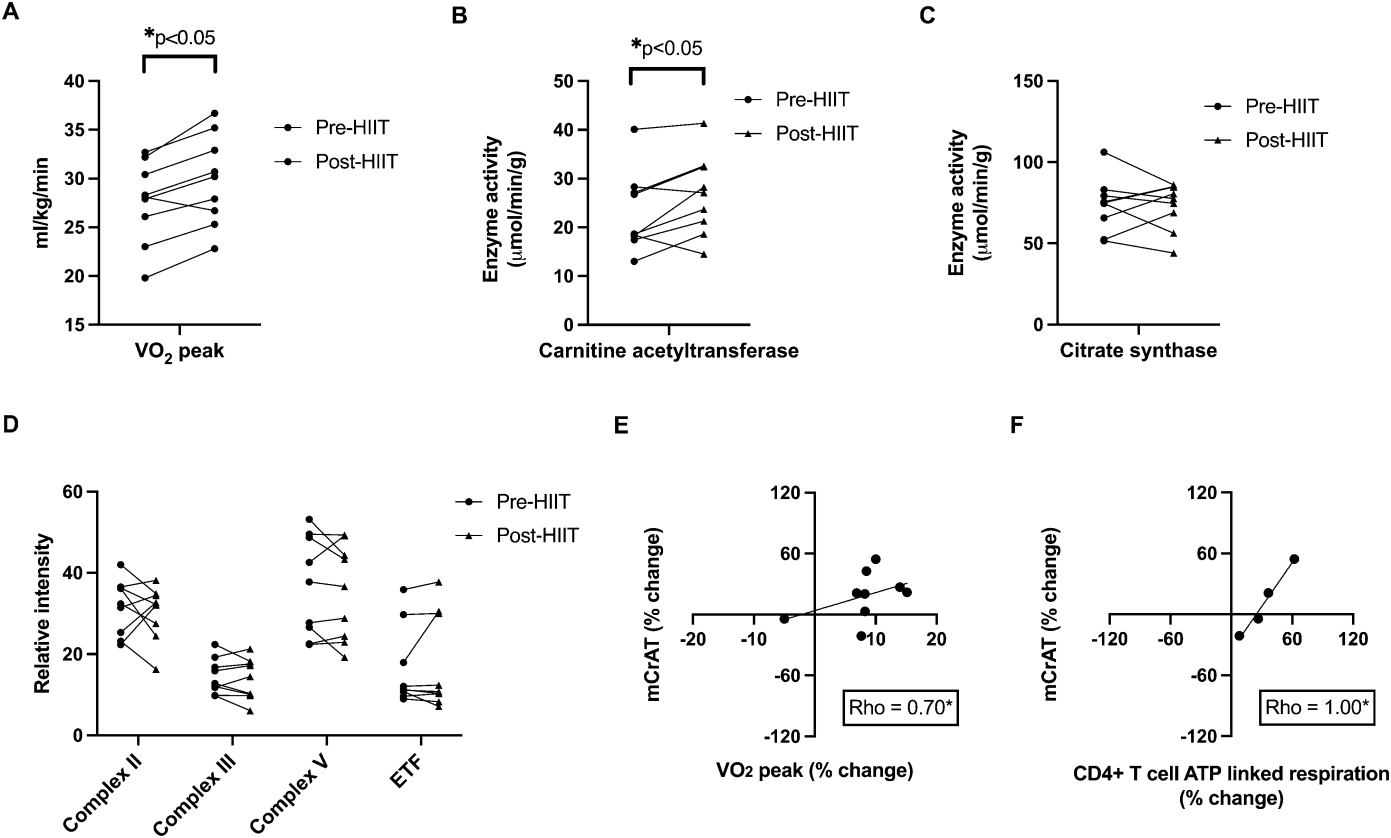
HIIT increases RA skeletal muscle carnitine acetyltransferase enzyme activity in association with increased cardiorespiratory fitness and changes in CD4 + T cell oxidative function. Graphs show individual rheumatoid arthritis (RA) participant (subgroup n = 9) (**A**) cardiorespiratory fitness (relative VO_2_; ml/kg/min), skeletal muscle (**B**) carnitine acetyltransferase (mCrAT) and (**C**) citrate synthase enzyme activity (μmol/min/g) before (Pre-HIIT) and after (Post-HIIT) high-intensity interval training (HIIT). (**D**) Graphs show individual RA participant protein expression of mitochondrial complexes II, III, and V and electron transfer flavoprotein (ETF) Pre-HIIT and Post-HIIT. (**E**) Scatter plot depicts relationship between percent change in RA mCrAT enzyme activity (y-axis) and percent change and percent change in relative peak VO_2_ (ml/kg/min) (x-axis) following HIIT. (**F**) Scatter plot depicts relationship between percent change in RA mCrAT enzyme acitivity (y-axis) and percent change and percent change in peripheral CD4 + T cell ATP linked respiration (y-axis) following HIIT. *p < 0.05 for paired t-tests and Spearman correlations.

Skeletal muscle mitochondrial oxidative enzyme function is critical for supporting aerobic physical activity and, in non-RA populations, significantly improves following aerobic exercise training^30,31^. It is unclear if patients with RA, who have skeletal muscle deficits in mitochondrial oxidative function, will show improved mitochondrial responses following exercise training. To address this question, we investigated the relationship between HIIT and muscle mitochondrial enzyme activity, and in turn, its relationship with changes in skeletal muscle metabolism and circulating T cell oxidative function. Using available RA skeletal muscle tissue from stored *vastus lateralis* biopsy samples from patients in this study (n = 9 from the larger cohort of 12 RA participants), we found that the activity of the mitochondrial enzyme carnitine acetyltransferase (CrAT) significantly increased following HIIT (pre-HIIT mean = 23.12 μmol/min/g, SD = 8.24; post-HIIT mean = 26.65 μmol/min/g, SD = 8.20, p = 0.04) (Fig. 3B). However, our results showed no changes following HIIT in the enzyme activity of mitochondrial citrate synthase (CS) (pre-HIIT mean = 73.68 μmol/min/g, SD = 16.57; post-HIIT mean = 73.09 μmol/min/g, SD = 14.40, p = 0.90) (Fig. 3C) and the protein expression of mitochondrial complexes II, IV, and V and electron transfer flavoprotein (p > 0.05 for all) (Fig. 3D). Unexpectedly, baseline RA skeletal muscle activities of CrAT and CS were not strongly correlated (rho = 0.53, p = 0.14); similarly, the percent (%) change in CrAT following HIIT was not correlated with % change in CS (rho = 0.07, p = 0.87). Notably, increases in RA muscle CrAT enzyme activity were associated with both increases in cardiorespiratory fitness (rho = 0.70, p = 0.04; Fig. 3E) and changes in peripheral CD4 + T cell ATP-linked respiration (rho = 1.00, p < 0.001; Fig. 3F) following HIIT.

### Changes in RA T cell oxidative metabolism associate with the skeletal muscle acylcarnitine metabolite profile

To inform our immune cell metabolism and muscle mitochondrial function analyses and interrelationships with cardiorespiratory fitness, we evaluated HIIT-induced changes in RA plasma amino acids, acylcarnitines, and non-esterified fatty acids and skeletal muscle organic acids, amino acids, and acylcarnitines. Plasma acylcarnitine C8:1 (mean percent increase = 44.3%, SD 50.5, p = 0.02 without multiple testing correction) was the only RA plasma metabolite to significantly change with HIIT (Supplemental Fig. 2). Skeletal muscle succinate—which increases in association with improved insulin sensitivity following exercise training in non-RA populations^32^—was the only RA skeletal muscle metabolite to significantly change with HIIT (mean percent increase = 73.9%, SD = 94.8, p = 0.03 without multiple testing correction) (Supplemental Fig. 3). Changes in RA plasma and skeletal muscle metabolomic profiles were not significantly associated with increases in cardiorespiratory fitness with the exception of an inverse relation between cardiorespiratory fitness and the concentration of muscle long-chain acylcarnitine C22:5 (n = 12; rho = − 0.59, p = 0.04 without multiple testing correction).

**Figure 4:**
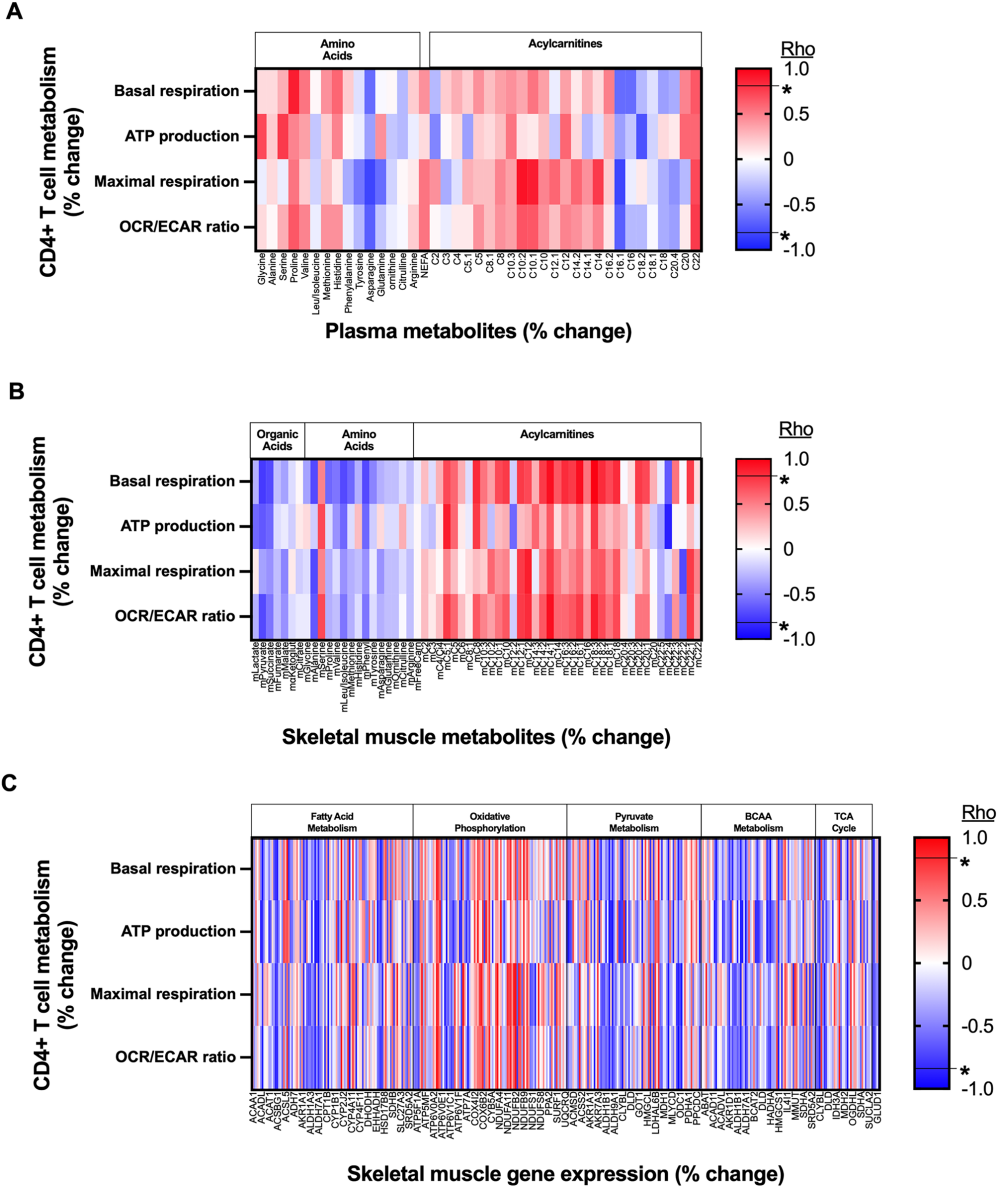
Changes in RA T cell respiration associate with changes in skeletal muscle acylcarnitine concentrations and increases in oxidative metabolism gene transcripts. (**A**) Heat map depicts correlations (Spearman’s rho) between percent change in rheumatoid arthritis (RA) peripheral CD4 + T cell respiration with plasma metabolites following high-intensity interval training (HIIT) (subgroup n = 6). (**B**) Heat map depicts correlations between percent change in RA peripheral CD4 + T cell respiration with skeletal muscle metabolites following HIIT (subgroup n = 6). (**C**) Heat map depicts correlations between percent change in RA peripheral CD4 + T cell respiration with skeletal muscle RNA with molecular relationships to carnitine acetyltransferase pathways (subgroup n = 6). *p < 0.05 (without multiple testing correction) for −0.8 ≤ rho ≤ 0.8. m Muscle, C Acylcarnitine.

We performed additional correlation analyses to better understand connections between CD4 + T cells with plasma and skeletal muscle metabolic changes following HIIT. Changes in CD4 + T cell respiration (basal, ATP production, maximal, and OCR:ECAR ratio) were, in general, not strongly associated with changes in plasma metabolites (Fig. 4A). In contrast, changes in CD4 + T cell respiration (basal, ATP production, maximal, and OCR:ECAR ratio) were, in general, negatively correlated with changes in skeletal muscle organic acids and amino acids but positively correlated with changes in skeletal muscle medium chain acylcarnitines, which are derived as byproducts of fatty acid metabolism (Fig. 4B)^32^.

### Muscle gene expression profile confirms an association between changes in T cell and skeletal muscle oxidative metabolism pathways

Since exercise training-related changes in RA CD4 + T cell respiration were strongly associated with changes in skeletal muscle fat metabolism pathways (i.e., muscle CrAT enzyme activity and muscle medium chain acylcarnitine concentrations), we hypothesized that RA T cell respiration would also link to the skeletal muscle oxidative metabolism molecular profile and enlighten potential causal pathways. First, we identified 408 genes involved in tricarboxylic acid (TCA) cycle, fatty acid metabolism, oxidative phosphorylation, pyruvate metabolism, branched chain amino acid (BCAA) metabolism, and glutamine/glutamate metabolism using the Kyoto Encyclopedia of Genes and Genomes (KEGG) pathways. Second, in these 408 genes, we assessed HIIT-related RA skeletal muscle expression changes (Supplemental Table 1)^33^. Of 408 molecules assessed, 10 significantly increased (p < 0.05 without multiple testing correction, over-represented by 5 genes with function in BCAA metabolism) and 9 significantly decreased (p < 0.05 without multiple testing correction) following HIIT (Supplemental Table 2). Although muscle CS enzyme activity did not change, CS gene expression significantly increased following HIIT (fold change post-HIIT versus per-HIIT = 1.100; p = 0.04 without multiple testing correction). Additionally, changes in CD4 + T cell respiration were, in general, most strongly associated with increases in skeletal muscle genes which function in oxidative phosphorylation and fatty acid metabolism (Fig. 4C). For example, changes in RA CD4 + ATP-linked respiration following HIIT was highly correlated (rho > 0.90 or <  − 0.90; p < 0.001) with expression changes in 9 genes (5 positive-, 4 negative-correlations) involved in muscle fatty acid metabolism pathways. The 5 positively correlated (i.e., changes in T cell respiration associated with increased expression) skeletal muscle genes (ATP6V0D2 (V-type proton ATPase subunit d 2), COX6A1 (Cytochrome c oxidase subunit 6A1), CYP2F1 (Cytochrome P450 2F1), IDH3G (Isocitrate dehydrogenase subunit gamma), NDUFA5 (NADH dehydrogenase 1 alpha subcomplex subunit 5)) had functions predominantly in oxidative phosphorylation and the TCA cycle^34^. The 4 inversely correlated (i.e., changes in T cell respiration associated with reduced expression) skeletal muscle genes (ACACB (acetyl-CoA carboxylase 2), ALDH3A2 (aldehyde dehydrogenase family 3 member A2), ALDH7A1 (alpha-aminoadipic semialdehyde dehydrogenase), ATP6V1B1 (V-type proton ATPase subunit B kidney isoform)) had predominant functions in lipid storage and aldehyde conversion to fatty acids^34^. These results in total suggest that HIIT-induced improvements in cardiorespiratory fitness link to co-occurring changes in skeletal muscle and CD4 + T cell oxidative metabolic function in our cohort of patients with RA.

## Discussion

In RA, metabolic reprogramming—reduced oxidative metabolism—occurs in both T cells and skeletal muscle, contributing to increased immune cell tissue invasiveness, disease activity, and disability^9,12^. In healthy persons, both immune cell and muscle oxidative metabolism improve with exercise training^19–23^; these oxidative function gains suggest that in RA, exercise training could modify disease activity through metabolic reprogramming of immune cells and skeletal muscle. In this cohort of RA patients who completed 10 weeks of HIIT and improved inflammatory disease activity^18^, VO_2_ peak improved in 11 of 12 participants. The subgroup of 6 RA participants for which T cell metabolism was assessed included one that had an unusually strong negative cardiorespiratory response to HIIT. Interestingly, in this subgroup of RA participants, we found that HIIT-induced changes in cardiorespiratory fitness were associated with changes in both peripheral CD4 + T cell respiration and molecular markers of skeletal muscle oxidative metabolism. In addition, changes were related across systemic oxidative metabolism (*i.e.,* cardiorespiratory fitness), T cell respiration, muscle CrAT activity, plasma and muscle metabolic intermediates, and muscle gene expression. These apparent interconnections between whole body, skeletal muscle, and T cell metabolism highlight the potential for exercise training and improved cardiorespiratory fitness in RA treatment augmentation.

Altered RA naïve CD4 + T cell metabolism is linked to pro-inflammatory function and is characterized by reduced glycolysis and low oxidative ATP production^11,12^. In non-RA populations, naïve CD4 + T cells and anti-inflammatory T cell subsets (i.e., regulatory T cells) primarily use oxidative phosphorylation energy substrates^35^. With HIIT, changes in RA peripheral naïve CD4 + T cell numbers positively associate with changes in CD4 + T cell ATP-linked respiration. Given that healthy naïve CD4 + T cells are important for immune homeostasis and directing the appropriate response to foreign antigens, the ability of exercise to alter oxidative metabolism in RA CD4 + T cells may signify a shift toward better functioning, less inflammatory, and less immunosenescent T cell populations^28,36^. Further studies are needed to assess the impact of exercise training on the metabolic function of specific T cell subsets (such as naïve, memory, Th1, Th17, and regulatory T cells).

Our finding that exercise-induced changes in RA fitness associate with changes in CD4 + T cell respiration has not been previously reported. In a healthy population, 8-weeks of aerobic exercise training increases peripheral blood mononuclear cell (PBMC) proteins important for mitochondrial biogenesis^37^. However, in young healthy men, a short HIIT program of two weeks did not impact PBMC respiration^38^. Taken together, these data suggest that the impact of exercise training on immunometabolism may vary across different study populations and PBMC compositions depending on baseline abnormalities. Further, the effect of the duration and intensity of exercise training on immune cell metabolism remains an open question.

Physical inactivity is a risk factor for the development of RA^13^, suggesting that exercise intolerance may be an important factor contributing to the loss of immune tolerance. While the pathways linking physical fitness with immune function in RA are unclear, physical activity and exercise have substantial immunoregulatory effects in non-RA populations. For example, acute strenuous exercise decreases peripheral CD4 + Th1 cells relative to Th2 cells in association with increases in circulating IL-10 and IL-6 levels^39,40^. Physical activity drives skeletal muscle release of IL-6; skeletal muscle-derived IL-6 then leads to increased CD4 + T cell IL-10 production and a net anti-inflammatory effect^15,41^. Lifelong exercise also leads to increased peripheral regulatory T cells, increased systemic transforming growth factor beta, decreased systemic IL-6 levels, and an anti-inflammatory skeletal muscle profile^42,43^. Thus, habitual physical activity may tip the balance towards an anti-inflammatory state via adaptations specifically in skeletal muscle, contributing to a decreased risk of chronic inflammation and autoimmunity^44^.

In our study, RA skeletal muscle metabolic adaptations to HIIT were marked by increased activity of the mitochondrial enzyme CrAT and alterations in the oxidative metabolism transcriptomic profile. Increases in skeletal muscle CrAT activity were associated with increased cardiorespiratory fitness. Further, changes in skeletal muscle CrAT activity, medium chain acylcarnitine concentrations (which reflect changes in flux through fatty acid beta-oxidation), and genes with function in fat oxidation and oxidative phosphorylation were associated with changes in CD4 + T cell respiration. Of note, skeletal muscle CrAT activity is important for high intensity exercise performance in mice^31^; whereas, low muscle CrAT activity is observed in the contexts of aging, obesity, and diabetes^45–47^. CrAT interconverts acetyl-CoA and acetylcarnitine and thereby buffers the mitochondrial acetyl group pool^31^, which in turn can impact oxidative flux of carbons derived from both glucose and fatty acids. These findings raise the possibility that improvements in muscle acetyl CoA buffering capacity in RA may be an important factor in the mechanisms by which exercise training downregulates inflammation.

In contrast to CrAT, the other RA skeletal muscle mitochondrial protein activities analyzed in this study (CS, complexes II/III/V, and electron transfer flavoprotein) did not substantially change on average after exercise training. Surprisingly, despite their close functional relationship to muscle mitochondrial metabolism^48^, baseline enzyme activities of CS and CrAT were not strongly correlated (rho = 0.53; p = 0.14); and, changes in CS did not associate with changes in CrAT or cardiorespiratory fitness following HIIT. One explanation for these discordant relationships is that the short chain acyl CoA buffering function of CrAT (which can impact pyruvate dehydrogenase and fatty acid metabolic pathways) is more specifically targeted by the HIIT program performed. Given that muscle CS gene expression did significantly increase despite muscle CS enzyme activity being unchanged following HIIT, another possibility is that the duration of exercise training was insufficient to 1) change the levels of muscle CS and electron transport proteins or 2) impact mitochondrial biogenesis in an RA population with poor oxidative metabolic function at baseline.

Our current study expands upon previous work reporting that cardiorespiratory fitness improvements in patients with RA associate with peripheral increases in the anti-inflammatory cytokine interleukin (IL)-10^16^ and decreases in galectin-3, a marker of chronic inflammation and cardiovascular disease risk^17^. These associations among improvements in cardiorespiratory fitness, improvements in inflammation, and changes in immune cell and systemic metabolism highlight the potential for cardiorespiratory fitness as a critical target for modulating RA cardiometabolic risk. Further, analyses from the current study—regarding interconnections between changes in oxidative metabolism concurrently in skeletal muscle and immune cells following HIIT—support our hypothesis that altered RA skeletal muscle metabolism in the sedentary state may contribute to perpetuating immune activation. At the same time, exercise training can help to rewire metabolic signaling between those organ systems^18^.

The findings in this study should be considered in the context of a few key limitations. These limitations are primarily due to the challenges of completing complex, multi-level phenotyping in patients with RA. For example, changes in T cell metabolic parameters were not interpreted in light of any changes in immune function (e.g., cytokine production, proliferation, migration), an area for future research. Further, the primary analyses for this investigation evaluated associations, so causality could not be determined. Also, a key limitation is the small sample size. The size of the cohort in some analyses was further limited by the availability of only six paired lymphocyte and nine paired skeletal muscle tissue samples from subgroups, which were leftover after completion of initial primary analyses for the study herein^16,17^. Another limitation was the lack of an appropriate healthy control group (i.e., matched non-RA population that participated in HIIT program to better evaluate RA-specific phenomenon). Given the observational design of the study, RA participants were also not randomized to the HIIT program versus usual care (i.e., the preferred study design for determining the effects of a specific exercise intervention such as HIIT). The interpretation of these results must also take into account the differences in methodology for assessing mitochondrial function in immune cells and skeletal muscle. We chose these methods because of the technical limitations of laboratory analysis with the available samples. Extracellular flux analysis (which was used for immune cell phenotyping) is a widely accepted method for functionally assessing cellular respiration; however, only a limited number of skeletal muscle samples stored at −80 °C were available, which made it necessary to indirectly assess mitochondrial metabolism using enzyme activity assays and Western blot protein quantification. On the other hand, we were able to corroborate several important relationships between exercise training-related changes in T cell and skeletal muscle metabolism at multiple levels (gene transcript, metabolite, and protein).

In summary, we found in our pilot study of 12 patients with RA that 10 weeks of HIIT significantly improved cardiorespiratory fitness. In a subgroup of these RA patients, including one that had an unusual negative cardiorespiratory response to HIIT, changes in fitness associated with changes in both peripheral helper T cell respiration and molecular markers of skeletal muscle oxidative metabolism. Exercise training-related changes in RA helper T cell mitochondrial metabolism were also positively associated with 1) changes in RA circulating naïve helper T cells, and 2) molecular changes in oxidative metabolism in RA skeletal muscle. Taken together, these findings highlight the need for further in-depth study to identify whether exercise training—possibly through adaptations in skeletal muscle—can alter systemic immune cell metabolism. Importantly, these results also highlight the potential importance of promoting cardiorespiratory fitness as a means to improve overall cardiometabolic health in RA.

## Methods

### Study design and participants

Previously sedentary participants with RA (n = 12) underwent phlebotomy, skeletal muscle biopsies, and cardiopulmonary exercise testing before and after a 10-week HIIT exercise program^16,17^. All subjects with RA met the following inclusion criteria: (1) satisfied American College of Rheumatology/European League Against Rheumatism 2010 RA classification criteria and were either seropositive (positive test for rheumatoid factor and/or anti-CCP antibodies) or had joint erosions on hand radiographs^49^; (2) had no medication changes during the previous 3 months; (3) were taking prednisone in doses of 5 mg per day or less; and (4) were exercising less than 2 days per week prior to the study. Participants were excluded if they had diabetes mellitus or cardiovascular disease and if they were unable to walk unaided on a treadmill.

### Exercise intervention and acquisition of blood and tissue samples

Participants underwent supervised exercise sessions three times per week for 10 weeks with continuous heart rate monitoring as previously described^16,17^. All exercise was performed via treadmill walking; participants achevied higher intensities by increasing treadmill speed and grade. Briefly, during each session, participants completed a 5-min warm up, followed by 10 alternating high-intensity (80–90% heart rate reserve) and low-intensity (50–60% heart rate reserve) intervals (60–90 s each), and a 5-min cool-down. After initial primary analyses (n = 12) were completed^16,17^, we used all leftover stored samples obtained via phlebotomy (subgroup n = 6) and *vastus lateralis* needle biopsies (subgroup n = 9) obtained by the standard Bergstrom technique^22^ before and after 10 weeks of HIIT for current pilot study analyses.

### Outcome measures

The co-primary outcomes were changes in RA peripheral CD4 + T cell mitochondrial respiration and skeletal muscle fiber expression of select mitochondrial metabolic enzymes. Secondary outcomes were quantification of peripheral lymphocyte subsets; plasma and skeletal muscle organic acids, amino acids and acylcarnitines; and skeletal muscle gene expression profiles. Cardiorespiratory fitness was measured via maximal oxygen consumption during exercise (VO_2_ peak) assessment with graded treadmill exercise testing^16^.

### CD4 + T cell isolation

A total of 6 paired RA pre-HIIT and post-HIIT samples were used for peripheral immune cell analyses based on stored sample availability in the T cell subgroup. PBMCs were isolated from whole blood samples obtained in EDTA vacutainers before and after exercise training (within 2 h of phlebotomy) via density gradient centrifugation^16^. PBMCs stored and frozen in liquid nitrogen were thawed at 37 °C using the dropwise method in RPMI + 10% FCS + 1% penicillin–streptomycin + 1% L-glutamine (R10) + 50U/mL DNAse (R10/DNase) and counted for viability using trypan blue exclusion. PBMCs were resuspended at 1 × 10^6^ cells/mL in R10, before 1 × 10^5^ cells/tube removed for flow cytometry analyses. PBMCs were then plated with 100U/mL of rhIL-2 (StemCell Technologies) added; cells rested overnight at 37 °C/5% CO2. The following morning, non-adherent lymphocytes were removed and washed in phosphate-buffered saline + 2% bovine serum albumin + 1 mM EDTA (PBS/BSA), counted and viability checked. CD4 + T cells were then isolated using immunomagnetic negative selection, using manufacturer guidelines (StemCell Technologies). CD4 + T cell purity was > 95% for all samples as assessed by fluorescence-activated cell sorting flow cytometry using CD3 FITC and CD4 PE antibody labeling on a BD FACS Canto II analyzer. CD4 + T cells, were then washed and resuspended in R10 buffer containing 100U/mL rhIL-2 and activated by addition of 25µL/mL CD3/CD28 tetrameric antibody complexes (StemCell Technologies) for 3 days using manufacturer guidelines.

### CD4 + T cell mitochondrial respiratory function analysis

Following 3 days of activation, cells were removed, washed in R10, counted and checked for viability. Isolated peripheral CD4 + T cell OCR (pmol/minute) and ECAR (mpH/minute) were measured by a Seahorse XF96 extracellular flux analyzer (Agilent, Wilmington, DE) using the Cell Mito Stress Test Kit, using manufacturer guidelines, through the Duke University Seahorse Extracellular Flux Analyzer Core. Briefly, CD4 + T cells were plated in wells (2.5 × 10^5^ viable cells/well) containing Cell-Tak (BD Bioscience) for 35 min in a 5% CO_2_ incubator at 37 °C. Cells were briefly and gently spun, and the monolayer checked by microscope before addition of XF assay media (glucose (1 M), pyruvate (100 mM), glutamine (200 mM)) and incubated for 40 min in a 5% CO_2_ incubator. The following compounds were added to the appropriate ports on the sensor cartridge for a final concentration of: (A) oligomycin (1.5 µM), (B) carbonyl cyanide-4-(trifluoromethoxy) phenylhydrazone [FCCP (0.5 µM)], (C) Rot/AA (0.5 µM). Following calibration, the cell culture plate was added to the Seahorse analyzer for a 3-h assay. Measurements were acquired every 15 min, with three measurements completed before injection of each compound. Data were analyzed using Wave Desktop 2.6 software (Agilent). Primary outcomes reported are basal respiration (baseline OCR prior to addition of oligomycin minus non-mitochondrial OCR following addition of Rot/AA), ATP-linked respiration (baseline OCR minus non-mitochondrial OCR following addition of Rot/AA and proton leak OCR following addition of oligomycin), and maximal respiration (OCR following addition of FCCP minus non-mitochondrial OCR following addition of Rot/AA).

### Lymphocyte flow cytometry

PBMCs thawed on day one were washed twice in PBS + 1% BSA (PBS/BSA) and resuspended in 100µL PBS/BSA containing previously titrated combinations or singles of fluorescently conjugated antibodies against human 1 µg/mL CD3 Pacific Blue (Clone UCHT1; BD Bioscience), 1 µg/mL CD4 FITC (Clone OKT4; BioLegend), 0.2 µg/mL CD28 PE (Clone CD28.2; BioLegend), 1.5 µg/mL CCR7 APC (Clone G043H7; BioLegend), 0.5 µg/mL CD45RO APC-Cy7 (Clone UCHL1; BioLegend), 0.5 µg/mL CD45RA PE-CY7 (Clone HI100; BioLegend), 0.1 µg/mL CD56 PE (Clone HCD56; BioLegend), 1.5 µg/mL CD19 APC-Cy7 (Clone HIB19; BioLegend), and 5µL CD16 FITC (Clone B73.1; BD Biosciences). Cells were analyzed separately for T cells (CD3, CD4, CD28, CD45RA, CD45RO, and CCR7), NK cells (CD3, CD56, and CD16), and B cells (CD3 and CD19). Cells were incubated with antibodies for 30 min on ice in the dark before washing once in PBS/BSA and fixed with 1% formalin (Sigma Aldrich) for 20 min at RT in the dark. Cells were then washed and resuspended in 300 µL PBS/BSA and immediately analyzed on a BD FACS Canto II equipped with 3 lasers at the Duke Cancer Institute Flow Cytometry Core. All analyses were completed after acquisition using FCS Express v6 (DeNovo Software, CA).

### Skeletal muscle mitochondrial activity assays

A total of 9 paired RA pre-HIIT and post-HIIT samples—obtained from *vastus lateralis* via needle biopsies using standard Bergstrom technique after an overnight fast and between 24–48 h after last exercise bout—were used for skeletal muscle tissue analyses based on stored sample availability in the skeletal muscle subgroup^50^. Stored skeletal muscle samples frozen at −80 °C were powdered and diluted 20-fold in CelLytic MT lysis buffer with protease and phosphatase inhibitors. Tissue lysates were then hand homogenized and freeze-fractured and total protein concentrations were determined by BCA assay. Lysates were diluted in CelLytic MT at 2 mg/dL for activity assays. Activities of skeletal muscle mitochondrial metabolic enzymes CS and CrAT were assessed with activity assays as previously described^31,51–53^. We selected these enzymes because they correlate strongly with mitochondrial content and muscle health, increase after exercise training, and decrease with aging^31,51–53^. In brief, serially diluted triplicates using human samples were performed to ensure enzyme activity was in the linear range. For both CS and CrAT activity assays, 20 μL of sample was added to each well and technical triplicates were averaged for each sample. For CS assays, the rate of conversion of DTNB to TNB prior to adding 10 μL of 10 mM oxaloacetate was subtracted from the post-oxaloacetate rate to calculate the citrate synthase activity (μmol/min/g). For CrAT assays, the rate of DTNB to TNB prior to adding 10 μL of 200 mM L-carnitine was subtracted from the post-carnitine rate to calculate the carnitine acetyltransferase activity (μmol/min/g).

### Skeletal muscle mitochondrial protein Western blots

Skeletal muscle sample lysates for Western blotting were prepared as described for activity assays above. Expression levels for OXPHOS antibody cocktail (Abcam, ab110413, mouse, monoclonal) and electron transfer flavoprotein (Abcam, ab110316, mouse, monoclonal) were determined using standard Western blotting technique. In brief, aliquots were prepared at 1.5 mg/ml and 20 μl of sample per well. 30 μg protein of each sample was electrophoresed in BioRad Criterion TGX Stain Free precast gels and transferred to a BioRad nitrocellulose gel membrane. The membrane was first put through a reversible total protein stain using the Ponceau method. The membrane was then was blocked with fish gelatin blocking buffer, incubated with the primary antibody overnight rocking at 4° C, then stained with secondary 680 nm mouse antibody for 1 h. Each membrane was then imaged for stained protein quantification on LI-COR Odyssey CLx. The final readings were then normalized to the total protein levels per each well. Expression levels for complex I of the OXPHOS antibody cocktail are not reported due to staining intensity measurements below the minimum level of detection.

### Metabolomic profiling

Stored, frozen plasma and skeletal muscle samples from the previously completed study were used for metabolomic analyses^16^. Plasma amino acids, non-esterified fatty acids, and acylcarnitines, and skeletal muscle organic acids, amino acids, and acylcarnitines were analyzed using targeted mass spectrometry as previously described^9,32,54,55^.

### Skeletal muscle gene expression profiling

Stored skeletal muscle samples from RA *vastus lateralis* needle biopsies (n = 12), obtained before and after 10 weeks of HIIT as above, were used for gene expression profiling^17^. Illumina Human HT-12v4 Expression BeadChips were used to quantitate RNA for analyses. 20–30 mg of muscle tissue was homogenized per frozen sample. Biotinylated total RNA—prepared using the Illumina TotalPrep RNA amplification kit (Life Technologies, Grand Island, NY, USA) and hybridized to Human HT-12 BeadChips (Illumina, San Diego, CA, USA)—was used for RNA profiling^9^. A targeted set of genes were chosen for comparative analyses based molecular relationships to CrAT using Ingenuity Pathway Analysis (IPA, www.ingenuity.com) filtering for KEGG pathways^33^: TCA cycle (38 molecules), fatty acid metabolism (108 molecules), oxidative phosphorylation (111 molecules), pyruvate metabolism (90 molecules), BCAA metabolism (70 molecules), and glutamine/glutamate metabolism (4 molecules) (Supplemental Table 1).

### Statistics

Predicted effect sizes for this pilot were assessed based on previous studies which show a mean increase in skeletal muscle CS concentrations of about 50% (SD = 25%) across multiple patient populations after exercise training^53^. Using paired t-tests comparing 9 paired pre- and post-training RA samples, assuming a conservative improvement estimate in muscle CS activity of 25% after exercise training, we had 80% power to detect a significant effect size of 0.80. Paired t-tests were used for pre- and post-intervention comparisons. Spearman’s rank correlations were used to determine the strength of relationships between outcome variables at baseline and percent change ((post-exercise variable minus pre-exercise variable)/pre-exercise variable × 100). Considerations were to use Pearson’s R or Kendall’s Tau correlations for analyses given small sizes, however, no substantive effect on the results was identified using these different statistical tests. Differences in RA skeletal muscle RNA fold change after HIIT were assessed via analysis of covariance (ANCOVA). Data was analyzed using Partek Genomics Suite (Partek, Inc.; St. Louis, MO, USA) for gene expression relationships and SAS statistical software (v.9.4) for all other assessments. Figures were created using GraphPad Prism 8. P values less than 0.05 were considered statistically significant for all analyses. Secondary analyses (i.e., metabolics and gene expression) were performed without multiple testing correction due to the exploratory nature of these assessments and goal to support primary analyses and guide future in-depth study.

### Ethics approval and consent to participate

All participants gave written informed consent. The study was approved by the Duke University Medical Center Institutional Review Board (IRB no. Pro00064057) and conducted according to the Declaration of Helsinki principles.

## Supplementary Information

Supplementary Information.

## Data Availability

The datasets generated and/or analyzed for the present study are available from the corresponding author on reasonable request.
