# Supplementary material for: Rheumatoid arthritis T cell and muscle oxidative metabolism associate with exercise-induced changes in cardiorespiratory fitness
Source: Sci Rep. 2022 May 6;12:7450. doi: 10.1038/s41598-022-11458-4 (PMC9076829; doi:10.1038/s41598-022-11458-4)
Supplement: Supplementary file 1 — Supplementary Information. [file 41598_2022_11458_MOESM1_ESM.pdf]

Rheumatoid arthritis T cell and muscle oxidative metabolism associate with exercise-induced changes in cardiorespiratory fitness

Brian J. Andonian, MD, MHSc[1,2*], Alec Koss, BS[2], Timothy R. Koves, PhD, MBA[2], Elizabeth R. Hauser, PhD[2], Monica J. Hubal, PhD[3], David M. Pober, PhD[4], Janet M. Lord, PhD[5,6], Nancie J. MacIver, MD, PhD[7], E. William St Clair, MD[1], Deborah M. Muoio, PhD[2], William E. Kraus, MD[2], David B. Bartlett, PhD[2,8#], Kim M. Huffman, MD/PhD[1,2#]

[1]Division of Rheumatology and Immunology, Duke University School of Medicine, Durham, NC 27701, USA.

[2]Duke Molecular Physiology Institute, Duke University School of Medicine, Durham, NC, 22701 USA.

[3]Department of Kinesiology, Indiana University-Purdue University Indianapolis School of Health & Human Sciences, Indianapolis, IN, 46202, USA

[4]Phastar Inc, Cambridge, MA, USA

[5]MRC-Versus Arthritis Centre for Musculoskeletal Ageing Research, Institute of Inflammation and Ageing, University of Birmingham, UK.

[6]NIHR Birmingham Biomedical Research Centre, University Hospital Birmingham and University of Birmingham, Birmingham, UK.

[7]Department of Pediatrics, University of North Carolina, Chapel Hill, NC, 27514 USA

[8]Faculty of Health and Medical Sciences, University of Surrey, Guildford, UK

*Corresponding author and lead contact: brian.andonian@duke.edu

[#]These authors contributed equally

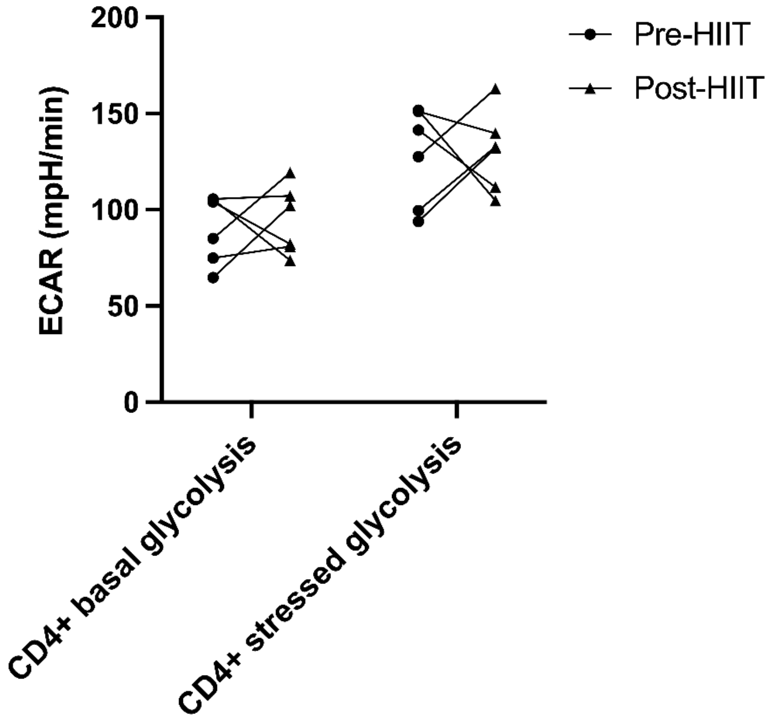

Supplemental Figure 1. Changes in rheumatoid arthritis CD4+ T cell glycolytic function following high-intensity interval training. Graphs show changes from before (Pre-HIIT) to after (Post-HIIT) high-intensity interval training (HIIT) in individual rheumatoid arthritis (RA) participant peripheral CD4+ T cell basal and stressed (peak value during Seahorse Mito Stress Test) extracellular acidification rate (ECAR). $p>0.05$ for Pre-HIIT versus Post-HIIT paired t tests.

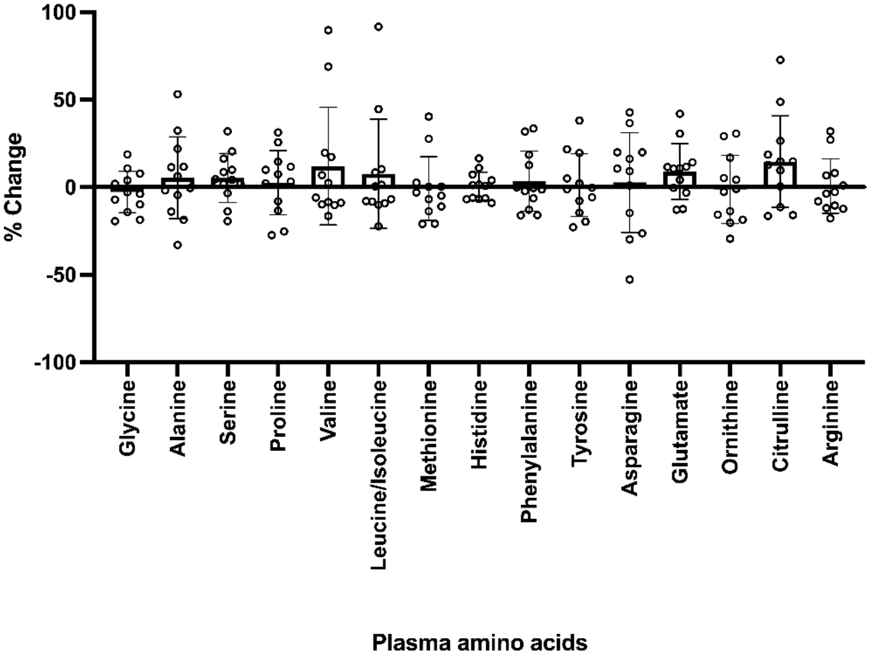

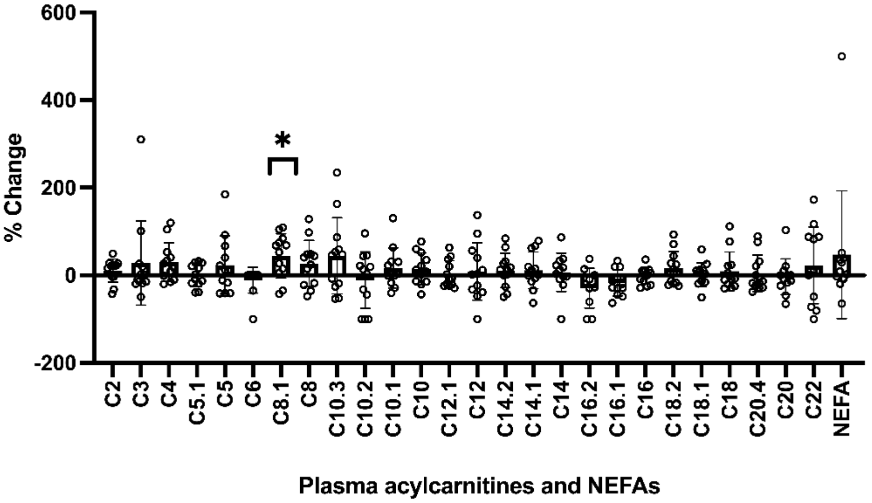

Supplemental Figure 2. Rheumatoid arthritis plasma metabolite changes after high-intensity interval training. A) Graphs show percent changes in individual rheumatoid arthritis (RA) plasma amino acid concentrations following high-intensity interval training (HIIT) data as dot plots with means (box) and SD (whiskers). B) Graphs show percent changes in RA plasma acylcarnitine and non-esterified fatty acid concentrations following HIIT data as dot plots with means (box) and SD (whiskers). * $p<0.05$ (without multiple testing correction) for RA plasma metabolite concentrations before HIIT versus after HIIT paired t tests.
m Muscle, c Acylcarnitine, NEFA Non-esterified fatty acids

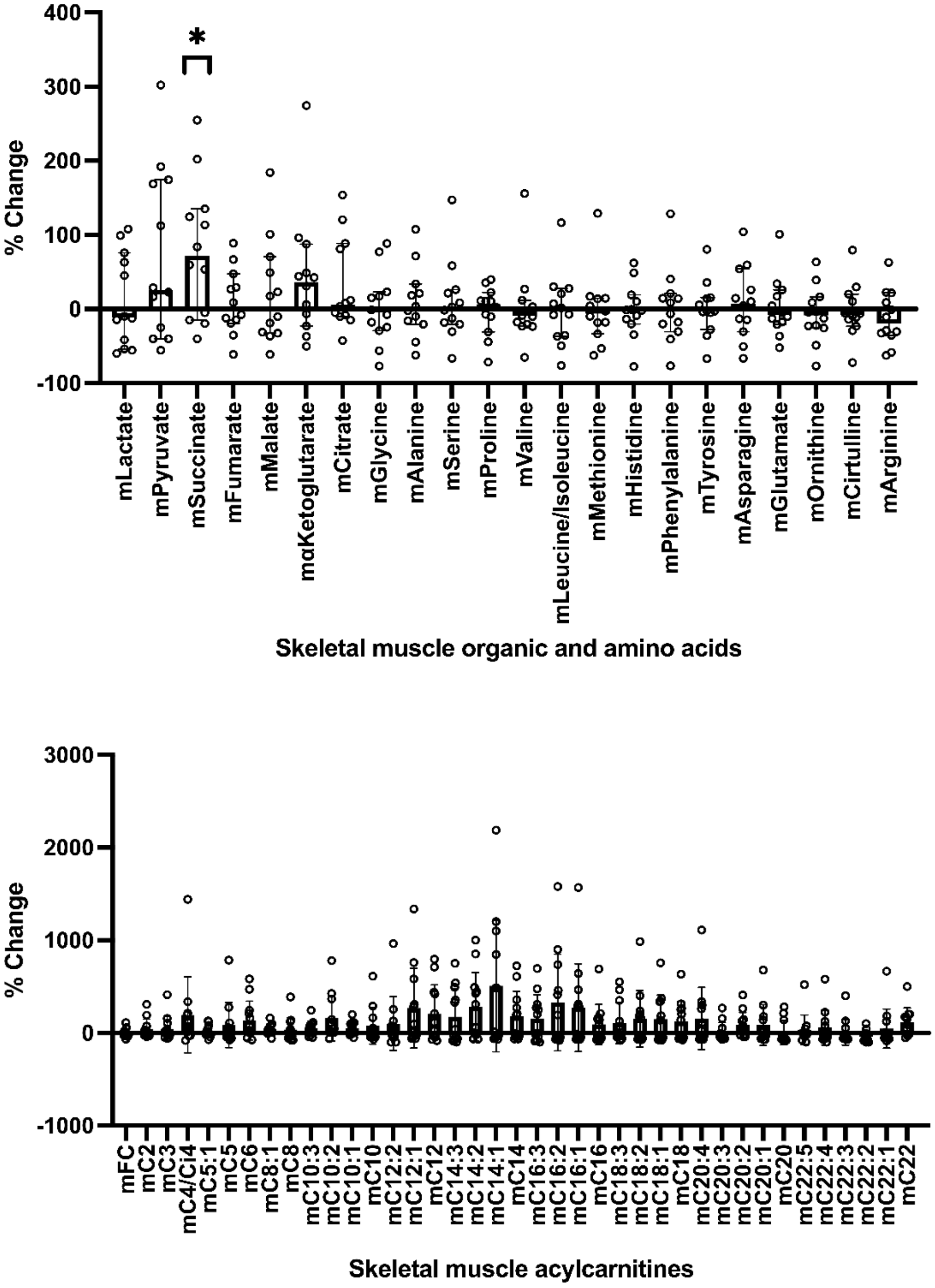

Supplemental Figure 3. Rheumatoid arthritis skeletal muscle metabolite changes after high-intensity interval training. A) Graphs show percent changes in individual rheumatoid arthritis (RA) skeletal muscle organic acid and amino acid concentrations following high-intensity interval training (HIIT) data as dot plots with means (box) and SD (whiskers). B) Graphs show percent changes in individual RA skeletal muscle acylcarnitine concentrations following HIIT data as dot plots with means (box) and SD (whiskers). *$p<0.05$ (without multiple testing correction) for RA skeletal muscle metabolite concentrations before HIIT versus after HIIT paired t test. m Muscle, C Acylcarnitine

Supplementary Table 1. KEGG molecule list for gene expression analyses

KEGG pathway category	Molecules
Fatty Acid Metabolism	ACAA1, Acaa1b, ACAA2, ACAD10, ACAD11, ACAD8, ACADL, ACADM, ACADS, ACADSB, ACADVL, ACAT1, ACAT2, ACOX1, ACOX2, ACOX3, ACSBG1, ACSBG2, ACSL1, ACSL3, ACSL4, ACSL5, ACSL6, ADH1A, ADH1B, ADH1C, ADH4, ADH5, ADH6, Adh6a, ADH7, ADHFE1, AKR1A1, AKR1D1, ALDH1A1, ALDH1A2, ALDH1A3, ALDH1B1, ALDH2, ALDH3A1, ALDH3A2, ALDH4A1, ALDH7A1, ALDH9A1, Aldr5, AUH, CPT1A, CPT1B, CPT1C, CPT2, CYP19A1, CYP1A1, CYP1A2, CYP1B1, Cyp2b23, CYP2B6, Cyp2c23, Cyp2c70, CYP2C8, Cyp2d1/Cyp2d5, Cyp2d22, Cyp2d26, CYP2E1, CYP2F1, CYP2J2, Cyp2j5, CYP2S1, CYP2U1, CYP3A4, CYP3A5, CYP4A11, Cyp4a14, CYP4A22, CYP4B1, CYP4F11, CYP4F12, CYP4F2, CYP4F8, CYP4X1, CYP4Z1, CYP51A1, DHODH, DHRS9, ECHS1, ECI1, ECI2, EHHADH, GCDH, HADH, HADHA, HSD17B10, HSD17B4, HSD17B8, IVD, POR, SDHA, SDHB, SDHD, SDS, SLC27A1, SLC27A2, SLC27A3, SLC27A4, SLC27A5, SLC27A6, SRD5A1, SRD5A2, SRD5A3
Oxidative Phosphorylation	ATP12A, ATP4A, ATP4B, ATP5F1A, ATP5F1B, ATP5F1C, ATP5F1D, ATP5MC3, ATP5MF, ATP5PB, ATP5PF, ATP6AP1, ATP6V0A1, ATP6V0A2, ATP6V0A4, ATP6V0B, ATP6V0D1, ATP6V0D2, ATP6V0E1, ATP6V0E2, ATP6V1A, ATP6V1B1, ATP6V1B2, ATP6V1C1, ATP6V1C2, ATP6V1D, ATP6V1E1, ATP6V1E2, ATP6V1F, ATP6V1G1, ATP6V1G2, ATP6V1G3, ATP6V1H, ATP7A, COX10, COX11, COX15, COX4I1, COX4I2, COX5A, COX6A1, COX6A2, COX6B1, COX6B2, COX7A1, COX7B, COX7B2, Cox7b2, COX8A, Cox8b, CYB5A, LHPP, MT-ATP6, mt-Atp8, MT-ATP8, MT-CO1, MT-CO2, MT-CO3, MT-CYB, MT-ND1, MT-ND2, MT-ND3, MT-ND4, MT-ND4L, MT-ND5, MT-ND6, NDUFA1, NDUFA11, NDUFA12, NDUFA13, NDUFA2, NDUFA3, NDUFA4, NDUFA4L2, NDUFA5, NDUFA6, NDUFA8, NDUFA9, NDUFB1, NDUFB10, NDUFB11, NDUFB2, NDUFB5, NDUFB6, NDUFB7, NDUFB8, NDUFB9, NDUFC1, NDUFC2, NDUFS1, NDUFS2, NDUFS4, Ndufs6, NDUFS6, NDUFS7, NDUFS8, NDUFV1, NDUFV2, NDUFV3, PPA1, PPA2, SDHA, SDHB, SDHC, SDHD, SURF1, TCIRG1, UQCRC1, UQCRC2, UQCRFS1, UQCRQ
Pyruvate Metabolism	ACACA, ACACB, ACAT1, ACAT2, ACMSD, ACOT12, ACSL1, ACSL3, ACSS1, ACSS2, ACSS3, ACYP1, ACYP2, ADH4, AKR1A1, AKR1B1, Akr1b7, AKR1C1/AKR1C2, AKR1C3, AKR1C4, AKR7A2, AKR7A3, ALDH1A1, ALDH1A2, ALDH1A3, ALDH1B1, ALDH2, ALDH3A1, ALDH3A2, ALDH4A1, ALDH7A1, ALDH9A1, Aldr2, Aldr3, Aldr4, AMD1, AZIN2, BCKDHA, BCKDHB, CLYBL, CSAD, DBT, DDC, DLAT, DLD, ECHDC1, GAD1, GAD2, GLO1, GOT1, GRHPR, HADHA, HAGH, HDC, HMGCL, HMGCLL1, HOGA1, ILVBL, LDHAL6A, LDHAL6B, LDHB, LDHC, LDHD, LIPF, LOC100362738, MDH1, MDH2, ME1, ME2, ME3, MLYCD, MVD, MYO5B, NANS, NPL, ODC1, PAICS, PC, PCK1, PCK2, PDHA1, PDHB, PISD, PKLR, PKM, PPCDC, UMPS, URAD, UROD, UXS1
Valine, Leucine, Isoleucine Metabolism	ABAT, ACAA1, Acaa1b, ACAA2, ACAD10, ACAD11, ACAD8, ACADL, ACADM, ACADS, ACADSB, ACADVL, ACAT1, ACAT2, ADH7, AGXT2, AKR1D1, ALDH1A1, ALDH1A2, ALDH1A3, ALDH1B1, ALDH2, ALDH3A1, ALDH3A2, ALDH4A1, ALDH6A1, ALDH7A1, ALDH9A1, AOX1, Aox3, Aox4, AUH, BCAT1, BCAT2, BCKDHA, BCKDHB, DBT, DHODH, DLD, ECHS1, EHHADH, GCDH, HADH, HADHA, HIBADH, HIBCH, HMGCL, HMGCLL1, HMGCS1, HMGCS2, HSD17B10, HSD17B4, HSD17B8, IL4I1, IVD, Lao1, MCCC1, MCCC2, MCEE, MMUT, OXCT1, PCCA, PCCB, SDHA, SDHB, SDHD, SDS, SRD5A1, SRD5A2, SRD5A3
TCA Cycle	ACAA1, Acaa1b, ACAA2, ACAD10, ACAD11, ACAD8, ACADL, ACADM, ACADS, ACADSB, ACADVL, ACAT1, ACAT2, ACOX1, ACOX2, ACOX3, ACSBG1, ACSBG2, ACSL1, ACSL3, ACSL4, ACSL5, ACSL6, ADH1A, ADH1B, ADH1C, ADH4, ADH5, ADH6, Adh6a, ADH7, ADHFE1, AKR1A1, AKR1D1, ALDH1A1, ALDH1A2, ALDH1A3, ALDH1B1, ALDH2, ALDH3A1, ALDH3A2, ALDH4A1, ALDH7A1, ALDH9A1, Aldr5, AUH, CPT1A, CPT1B, CPT1C, CPT2, CYP19A1, CYP1A1, CYP1A2, CYP1B1, Cyp2b23, CYP2B6, Cyp2c23, Cyp2c70, CYP2C8, Cyp2d1/Cyp2d5, Cyp2d22, Cyp2d26, CYP2E1, CYP2F1, CYP2J2, Cyp2j5, CYP2S1, CYP2U1, CYP3A4, CYP3A5, CYP4A11, Cyp4a14, CYP4A22, CYP4B1, CYP4F11, CYP4F12, CYP4F2, CYP4F8, CYP4X1, CYP4Z1, CYP51A1, DHODH, DHRS9, ECHS1, ECI1, ECI2, EHHADH, GCDH, HADH, HADHA, HSD17B10, HSD17B4, HSD17B8, IVD, POR, SDHA, SDHB, SDHD, SDS, SLC27A1, SLC27A2, SLC27A3, SLC27A4, SLC27A5, SLC27A6, SRD5A1, SRD5A2, SRD5A3
Glutamate and Glutamine Metabolism	GLS, GLS2, GLUD1, GLUD2

Supplementary Table 2. Significant changes in rheumatoid arthritis skeletal muscle gene expression following high-intensity interval training ($p<0.05$)

Gene ID	Gene name	KEGG pathway	Fold change (Post vs Pre)	p-value (ANCOVA)
Molecules significantly increased following high-intensity interval training				
BCKDHB	2-oxoisovalerate dehydrogenase subunit beta	Pyruvate Metabolism, Valine/Leucine/Isoleucine Metabolism	1.033	0.011
HSD17B10	3-hydroxyacyl-CoA dehydrogenase type-2	Fatty Acid Metabolism, Valine/Leucine/Isoleucine Metabolism	1.121	0.012
NDUFB6	NADH dehydrogenase [ubiquinone] 1 beta subcomplex subunit 6	Oxidative Phosphorylation	1.137	0.012
ACSL1	Long-chain-fatty-acid--CoA ligase 1	Fatty Acid Metabolism, Pyruvate Metabolism	1.161	0.021
BCAT2	Branched-chain-amino-acid aminotransferase	Valine/Leucine/Isoleucine Metabolism	1.081	0.030
ATP5MF	ATP synthase subunit f	Oxidative Phosphorylation	1.136	0.033
PKLR	Pyruvate kinase	Pyruvate Metabolism	1.033	0.033
HSD17B8	Estradiol 17-beta-dehydrogenase 8	Fatty Acid Metabolism, Valine/Leucine/Isoleucine Metabolism	1.089	0.036
ATP6V1F	V-type proton ATPase subunit F	Oxidative Phosphorylation	1.172	0.045
Molecules significantly decreased following high-intensity interval training				
ATP4B	Potassium-transporting ATPase subunit beta	Oxidative Phosphorylation	-1.030	0.001
OGDH	2-oxoglutarate dehydrogenase	TCA cycle	-1.033	0.003
LDHC	L-lactate dehydrogenase C chain	Pyruvate Metabolism	-1.043	0.009
ALDH6A1	Methylmalonate-semialdehyde dehydrogenase	Valine/Leucine/Isoleucine Metabolism	-1.084	0.028
ALDH9A1	4-trimethylaminobutyraldehyde dehydrogenase	Fatty Acid Metabolism, Pyruvate Metabolism, Valine/Leucine/Isoleucine Metabolism	-1.091	0.012
CYP4Z1	Cytochrome P450 4Z1	Fatty Acid Metabolism	-1.030	0.023
ATP6V0A2	V-type proton ATPase 116 kDa subunit a2	Oxidative Phosphorylation	-1.036	0.035
COX6B2	Cytochrome c oxidase subunit 6B2	Oxidative Phosphorylation	-1.045	0.039
TCIRG1	V-type proton ATPase 116 kDa subunit a3	Oxidative Phosphorylation	-1.043	0.040
